# Associations between Digital Health Intervention Engagement and Dietary Intake: A Systematic Review

**DOI:** 10.3390/nu13093281

**Published:** 2021-09-20

**Authors:** Tessa Delaney, Matthew Mclaughlin, Alix Hall, Sze Lin Yoong, Alison Brown, Kate O’Brien, Julia Dray, Courtney Barnes, Jenna Hollis, Rebecca Wyse, John Wiggers, Rachel Sutherland, Luke Wolfenden

**Affiliations:** 1 School of Medicine and Public Health, University of Newcastle, Callaghan, NSW 2308, Australia; Matthew.Mclaughlin1@health.nsw.gov.au (M.M.); Alix.Ivers@health.nsw.gov.au (A.H.); Serene.Yoong@health.nsw.gov.au (S.L.Y.); Alison.Brown7@health.nsw.gov.au (A.B.); Kate.OBrien@health.nsw.gov.au (K.O.); Julia.Dray@newcastle.edu.au (J.D.); Courtney.Barnes@health.nsw.gov.au (C.B.); Jenna.Hollis@health.nsw.gov.au (J.H.); Rebecca.Wyse@health.nsw.gov.au (R.W.); John.Wiggers@health.nsw.gov.au (J.W.); Rachel.Sutherland@health.nsw.gov.au (R.S.); Luke.Wolfenden@health.nsw.gov.au (L.W.); 2 Hunter New England Population Health, Wallsend, NSW 2287, Australia; 3 Hunter Medical Research Institute, New Lambton Heights, NSW 2305, Australia; 4 Priority Research Centre for Heath Behavior, University of Newcastle, Callaghan, NSW 2308, Australia; 5 Faculty of Health, Arts and Design, Swinburne University of Technology, Hawthorn, VIC 3122, Australia; 6 School of Psychology, University of Newcastle, Callaghan, NSW 2308, Australia

**Keywords:** engagement, adherence, digital health intervention, digital behavior change intervention, diet, nutrition, public health nutrition, digital technologies

## Abstract

There has been a proliferation of digital health interventions (DHIs) targeting dietary intake. Despite their potential, the effectiveness of DHIs are thought to be dependent, in part, on user engagement. However, the relationship between engagement and the effectiveness of dietary DHIs is not well understood. The aim of this review is to describe the association between DHI engagement and dietary intake. A systematic search of four electronic databases and grey literature for records published before December 2019 was conducted. Studies were eligible if they examined a quantitative association between objective measures of engagement with a DHI (subjective experience or usage) and measures of dietary intake in adults (aged ≥18 years). From 10,653 citations, seven studies were included. Five studies included usage measures of engagement and two examined subjective experiences. Narrative synthesis, using vote counting, found mixed evidence of an association with usage measures (5 of 12 associations indicated a positive relationship, 7 were inconclusive) and no evidence regarding an association with subjective experience (both studies were inconclusive). The findings provide early evidence supporting an association between measures of usage and dietary intake; however, this was inconsistent. Further research examining the association between DHI engagement and dietary intake is warranted.

## 1. Introduction

Poor diet is a leading preventable risk factor for non-communicable disease, accounting for 11 million deaths and 255 million disability-adjusted life years per annum [1]. Population surveys in Australia [2], the United Kingdom [3], and the United States [4] indicate that adults and children do not consume the recommended servings of fruit and vegetables and over-consume foods high in saturated fat, sugar, and salt. Efforts to improve population dietary intakes have been identified as a public health priority internationally [5]. 

The use of digital health interventions (DHI) has been recommended as a strategy to improve population dietary intake [6]. The World Health Organization refers to ‘digital health’ as the use of digital, mobile, and wireless technologies to support the achievement of health objectives and is both inclusive of m-health and e-health [7]. Digital health technologies may include mobile phones, portable computer tablets (e.g., iPads), web-based interventions, smartphone applications (apps) and wearable devices [8]. With 3.9 billion internet users and the potential to reach over 90% of the global population [9], DHIs, once developed, can be a cost-effective way of delivering interventions to large numbers of individuals and organizations in the population, and can be delivered with high fidelity and at low cost to a wide variety of populations, including disadvantaged groups [10]. 

Despite the promise of DHIs, systematic reviews evaluating the effectiveness of smartphone applications [11] and web-based interventions [12] provide mixed evidence on their effectiveness in improving dietary intake, with a lack of user engagement hypothesized as a limiting factor [13,14]. Engagement has recently been defined as both i) the extent of DHI usage, such as the amount, frequency, duration and depth of the DHI accessed, and ii) a subjective experience characterized by attention, interest and affect [15]. Whilst suggested to be important, the association between the characteristics of engagement and health behavior is not well understood [16]. As such, having a greater understanding of the relationship between engagement and dietary intake will likely provide an opportunity to optimize the impact of DHIs.

A 2011 systematic review of 33 studies examining the effect of engagement with web-based interventions and health outcomes found a positive relationship between DHI usage and fruit-and-vegetable intake, physical activity, weight management, and reductions in smoking and smokeless tobacco use [8]. The review found a positive relationship between DHI usage and improvement in dietary intake. However, the review included a narrow definition of engagement (e.g., focused on usage only), did not include a comprehensive search (e.g., included five keywords in the search strategy), and was restricted to web-based interventions, only, without considering other digital health technologies, such as m-health and smartphone applications. Furthermore, the systematic review identified just one study that assessed the association between DHI usage (logins) and dietary intake [8]. This randomized controlled trial of an online intervention found that more frequent website visits were associated with increased fruit-and-vegetable intake (*p* < 0.001) [17]. Since the 2011 review, there have been a large increase in research of DHIs targeting dietary intake [12,18]. This provides an opportunity to better understand the association between DHI engagement and dietary intake.

Therefore, the aim of the review is to systematically review the literature to describe the association between objective DHI engagement (both usage and subjective experience) and dietary intake.

## 2. Materials and Methods

This review was reported in accordance with the recommendations of the Joanna Briggs Institute for conducting systematic reviews of association [19] and was prospectively registered with the International Prospective Register for Systematic Reviews (PROSPERO) (CRD42018112189).

### 2.1. Search Strategy

A search of peer-reviewed literature was undertaken with the assistance of an experienced research librarian (DB) using the following four electronic databases: Embase, MEDLINE, PsycINFO, and Scopus. We considered records from inception to December 2019. There were no restrictions on the length of the study follow-up period or country of origin. Searches were restricted to the English language only. This review was conducted alongside another review aiming to describe the association between DHI engagement and physical activity and sedentary behaviour with findings reported in separate publications (PROSPERO CRD 42018110657). Therefore, ‘physical activity’ and ‘sedentary behavior’ search terms were also included in the search and the results reported elsewhere [20]. We used modified versions of published search filters and used Medical Subject Headings (MeSH) or free text words for physical activity [21], dietary intake [22], DHI engagement [15] and DHIs [15,23,24]. The search terms used were developed under the guidance of the research librarian, and the Medline search strategy was adapted for each database. Full details of the search strategy can be found in Supplementary File S1. Electronic bibliographic database searches were supplemented with hand searching of targeted journals and grey literature searches. Specifically, we conducted hand searching of all publications from January 2016 to December 2019 in the journals: Journal of Medical Internet Research, JMIR mHealth and uHealth, JMIR Medical Informatics and JMIR Public Health and Surveillance. We conducted grey literature searches in ‘Google.com/ncr’ search engine and used the search terms “Diet” AND “Engagement” AND “Digital Health Intervention” and screened the first 200 citations for relevance. We screened reference lists of similar systematic reviews of DHI engagement [8,15] and contacted authors of included studies for other potentially relevant studies. 

### 2.2. Inclusion Criteria

#### 2.2.1. Types of Studies

We included study designs that quantitatively examined an association between a measure of engagement with a DHI and any measure of dietary intake. Specifically, study designs could have included retrospective, prospective (e.g., randomized controlled trials, cohort studies), cross-sectional, before and after studies and interrupted time series studies. Engagement was defined as both the extent of the usage of the program (e.g., number of logins, time on site and activities completed) as well as the subjective experience, including measures of attention, interest, and affect [15]. DHIs were defined as the use of digital, mobile, and wireless technologies to support the achievement of health objectives. This was inclusive of both m-health and e-health. DHIs included, but were not limited to, portable computer tablets (e.g., iPads), web-based interventions and smartphone applications (apps) [7]. 

#### 2.2.2. Types of Participants

We included studies undertaken with adult (≥18 years defined by the mean age of the study sample at baseline) users of a DHI targeting dietary intake. Studies of participants that had access to a DHI and engaged with the DHI were eligible. As were studies targeting children or adolescents via an adult (parent or caregiver) use of a DHI, as long as the individual accessing the DHI was 18 years or above.

#### 2.2.3. Exposure (Independent Variable)

We included quantitative studies reporting any measure of engagement with a DHI, defined as the extent of usage (e.g., number of logins, time on site and activities completed) or the subjective experience of users (e.g., measures of attention, interest and affect, including but not limited to, enjoyment, satisfaction and user experience) as defined by Perski et al. [15]. Engagement could be collected by the DHI (e.g., usage analytics such as number of page logins, time spent online and the amount or type of intervention content used during the intervention period), observation (e.g., eye tracking), surveys of DHI users or other quantitative methods. Examples of measurement of engagement could include the frequency of use (typically measured by number of logins), the amount and/or duration of DHI use (typically measured by time on site), the type of content used (typically measured by activity completion e.g., use of an online tool), physiological measures (e.g., eye tracking, heart rate) and/or subjective experience of users such as quantitative measurement of attention, interest, affect, satisfaction, or usability of the DHI (typically measured via questionnaires e.g., ‘Systems Usability Scale’ [25], ‘Digital Behavior Change Intervention Engagement Scale’ [26], ‘User Engagement Scale’ [27], ‘eHealth Engagement Scale’ [28]) [14,29].

#### 2.2.4. Outcome (Dependent Variable)

We included studies reporting any measure of dietary intake, including, but not limited to, the intake of food or beverages (e.g., mean servings, proportion or quantity of fruit or vegetables); nutrients (e.g., mean kilojoules/calories, grams of nutrient of interest); nutritional value (e.g., healthy/less healthy); diet quality (e.g., diet quality index) or diet scores (e.g., Mediterranean Diet Score) [30]. Data could have been collected from food-frequency questionnaires, food diaries or 24-h recalls, participant surveys, direct observations, plate waste, or other quantitative sources. These may be reported in specific settings, or periods of the day (e.g., lunch) or as the whole day.

### 2.3. Exclusion Criteria

We excluded the following studies:

- Case studies, letters to the editor and non-empirical studies.
- Those which purposely sampled or recruited individuals on the basis of pre-existing health-related conditions, including chronic health conditions such as chronic pain, a chronic disease diagnosis, communicable disease or mental illness given our interest in generalizing the findings to general community samples.
- Those which targeted children (<18 years of age) through children’s use of a DHI.
- Those that used, in full or part, a non-DHI component (e.g., those with both face-to-face and digital intervention components). Studies that included a non-DHI component were excluded due to the difficulty in determining the effect between non-DHI and the DHI-exclusive intervention components on participant engagement.
- Studies that only reported qualitative assessments of engagement (e.g., focus groups).
- Studies that reported engagement with text messaging interventions with no other online component, e.g., CD-ROM and computer-based interventions not functioning in an online capacity;
- As this review was only focused on dietary intake, studies that targeted multiple health behaviors for prevention of chronic disease (e.g., sleep and diet, or diet and physical activity) were excluded to reduce the risk of other health behaviors confounding the association between engagement and dietary intake;
- Studies in which the full text was not available (e.g., where authors were unable to access full text online and/or after contact with the corresponding author).

### 2.4. Data Collection and Analysis

#### 2.4.1. Selection of Studies

After removal of duplicates, authors single screened titles and abstracts for potentially eligible studies using Covidence in two stages; titles and abstracts (CB, AB, MM) followed by full text (TD, MM, JH, CB). Review authors were not blind to author or journal information. The number of articles identified, screened, eligible, and included were recorded according to the Preferred Reporting Items for Systematic Reviews and Meta-Analyses (PRISMA) statement [31] (Supplementary File S2).

#### 2.4.2. Data Extraction and Management

Pairs of review authors (TD, MM) independently extracted data using a data extraction form adapted from the Cochrane Public Health Group Methods Manual and used previously by the research team [32]. Given the complexity of the review, a third author (AH) reviewed all data extracted and any disagreements in data extraction were resolved by the third author (AH). When study data were missing, we attempted to contact the authors to obtain the required information. The information extracted included:

1. Study characteristics, including authors’ names, publication year, overall study design, participant characteristics, study eligibility, and sample size.
2. Characteristics of the intervention, including type of DHI, length of exposure to the DHI, location of the DHI, target users of the DHI, and a description of the DHI including complexity and additional intervention strategies used.
3. Outcomes, including a description of the association, measures of dietary and engagement outcome and their validity; study design; analysis method used (including adjustments for confounds); magnitude of the association (odds ratio [OR] or regression coefficient or estimate; 95% confidence intervals [CI] or standard deviation [SD] or standard error [SE] and; *p*-values), the direction and favorability of effect; and information allowing quality assessment.

#### 2.4.3. Critical Appraisal

Pairs of review authors (JD, KO or AB, TD or TD, MM) assessed methodological quality of studies, independently, using the Newcastle—Ottawa Scale for cohort [33] or cross-sectional studies [34]. We defined cross-sectional as those studies using a single time-point of data for the dietary intake measure (e.g., follow-up), whereas cohort studies were those that used multiple time-points of data and calculated change over time (e.g., change from baseline to follow-up). The Newcastle—Ottawa Scale utilizes a star system to assess the methodological quality of cohort and cross-sectional studies. The cohort tool assigns a maximum of nine stars across three domains: (1) selection of study groups (up to four stars); (2) the comparability of these groups (up to two stars); and (3) assessment of outcomes (up to three stars). The cross-sectional tool assigns a maximum of ten stars across the same three domains: (1) selection of study groups (up to five stars), (2) the comparability of these groups (up to two stars), and (3) assessment of outcomes (up to three stars) (Supplementary File S3).

Within the cohort tool, the following items were assessed: representativeness of exposed cohort; selection of non-exposed cohort; ascertainment of exposure; outcome of interest; comparability of cohorts; assessment of outcome; length of follow-up and; adequacy of follow-up. When assessing the ‘adequacy of follow-up’, studies were required to have a minimum length of follow-up of 12 weeks to ensure that adequate time was allowed for reliable patterns of engagement to occur and be captured. Twelve-weeks was chosen based on current evidence [12] and a consensus process between two review authors (T.D., L.W.). Within the cross-sectional tool, for the item ‘the study controls for the most important factor’, we selected age and gender as the factors to control for confounding, given that these have been shown to be important prognostic factors influencing engagement [15]. Disagreements between assessments were resolved by discussion between the pairs of review authors (J.D., K.O. or A.B., T.D.) and, where required, by consulting a third review author.

#### 2.4.4. Data Synthesis and Analysis

Pooled quantitative synthesis was not possible due to high heterogeneity across the studies included in the review. An overview of all associations including direction, strength, and favorability, along with the characteristics of the included studies, are summarized, in full, in Table 1.

As meta-analysis was not possible, we used vote-counting methods, as recommended by Campbell et al. [35], to explore the direction of effect between each association of engagement (e.g., usage and subjective experience) and dietary intake (Table 4). Each association was assigned a ‘+’, ‘−‘, or ‘0′. Positive associations (of statistical significance) were denoted as ‘+’ and indicated that higher engagement was associated with improved dietary intake. Negative associations (of statistical significance) were denoted as a ‘−‘ and indicated that engagement was negatively associated with dietary intake. Non-significant associations in either direction were considered inconclusive associations and denoted with a ‘0′, as were studies wherein there were mixed findings reported. As the direction and strength of the association, irrespective of its statistical significance, are also recommended to be considered in descriptions of association, we also report the quantitative estimates in Table 1. 

For the vote count, studies were counted once for each engagement construct where they provided one or more measures of association. For example, if a study reported two associations of engagement (e.g., time on-site and logins) both would have been included in the vote count synthesis. If there were multiple tests of association reported using the same engagement measure and same dietary outcome in the one study (e.g., multiple associations reported for ‘time on site’ and fruit and vegetable intake), we used the following inclusion criteria to select the association of interest from each study for inclusion in the vote count:

- If a study had multiple associations using the same engagement measure and same dietary outcome, preference was given to the dietary outcome assessed using the instrument judged by the authors (in the absence of published reliability or validity data) to be most comprehensive. For example, if a study reported two associations including i) ‘time on website’ using an ‘all day’ fruit-and-vegetable screener and ii) ‘time on website’ using a ‘single-item’ fruit-and-vegetable screener, preference was given to the ‘all day screener’ as it is the more comprehensive outcome measure for fruit-and-vegetable intake.
- If multiple models were presented assessing the association between the same dietary outcome and same engagement measure (e.g., unadjusted and adjusted) we gave preference to the adjusted model.
- If multiple engagement measures were used and they all assessed the same type of engagement outcome (e.g., time on site) we selected the most complete and inclusive. For example, ‘total time on website’ was given preference to ‘time on a specific website feature’.

Studies were aggregated under the following standardized engagement variables: (i) usage, including ‘logins’, ‘time’, ‘composite usage’, or ‘activities completed’, or (ii) ’subjective experience’. Measures relating to logins or those that used logins as their data-collection method were categorized under ‘logins’. Any engagement measure that combined usage analytics into a single metric (‘time on site + logins’) were aggregated into a ‘composite-usage measure’. Measures that indicated completion of an activity within the DHI such as ‘recording vegetable intake’ were classified as ‘activities completed’. Finally, ‘engagement survey scores’ and ‘acceptability’ or ‘interest’ measures were aggregated into ‘subjective experience’. Dietary intake measures were categorized under the following standardized variable names: ‘fruit-and-vegetable intake’, ‘fruit intake’, ‘vegetable intake’, ‘calories from sugar-sweetened drinks’, or ‘other food groups’ on the basis of studies identified. Any measure of association that examined the relationship between food groups other than fruit and vegetables were classified as ‘other food group’ category.

## 3. Results

### 3.1. Search Results

**Figure 1:**
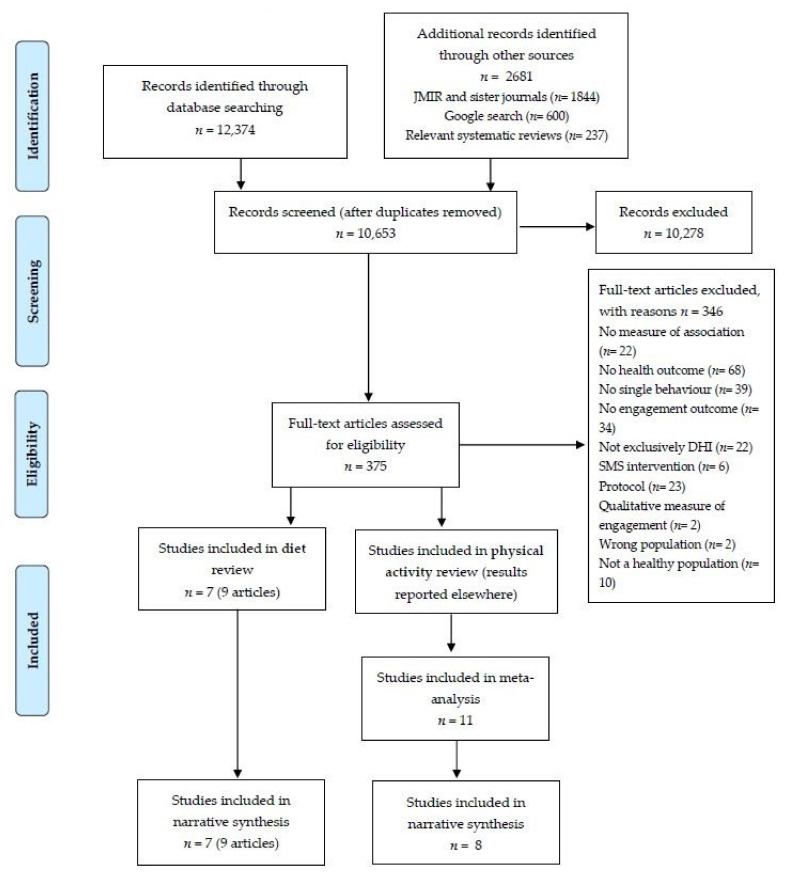
PRISMA flow diagram of included studies in the diet review.

The search resulted in 15,055 potentially relevant citations. Following the grey literature search and the removal of duplicates, 10,653 unique citations were retained and further screened for the review. Of this, we identified 375 full texts for screening. Following full-text screening, nine manuscripts from seven individual studies were included in the current review(Figure 1). Of the seven studies, five were web-based [17,36,37,38,39], one was an app [40], and one was an email-based intervention [41]. Three of the DHIs targeted fruit-and-vegetable intake [17,31,32,36], two targeted ‘healthy eating’ [33,34] and one targeted vegetable intake [35]. Six were cohort in design [17,36,37,38,39,41] and one was cross-sectional [40]. The majority of studies (*n* = 5) included usage [17,36,38,39,40] rather than subjective experience (*n* = 2) [37,41] as their engagement measure. No studies included both usage and subjective experience in measures of association. Four studies included ‘fruit-and-vegetable intake’ as their dietary measure [17,36,37,41], one study included ‘vegetable intake’ as their dietary measure [40] and the remaining two studies included multiple dietary intake measures, including ‘vegetable intake, ‘fruit intake’, intake from ‘other’ food groups, and ‘calories from sugar-sweetened drinks’ [38,39]. All seven studies used self-reported measures of dietary intake (e.g., FFQ). Of the 7 studies, six included validated tools to assess dietary intake [17,36,37,38,39,40]. The length of delivery of the DHI ranged from 3 weeks to 12 months. The study participants were predominantly females (range: 59 to 100%). The age of participants ranged from 18 to 65 years and the sample size ranged from 46 to 2513 participants (mean = 590). Characteristics of studies are described in Table 1.

### 3.2. Methodological Quality of Studies

Table 2 and Table 3 report the quality assessments for cohort studies and cross-sectional studies, respectively. Five cohort studies were rated ‘poor’ quality and one was rated as ‘good’ quality’, quality scores ranged from 3 to 6 out of a possible score of 9. The main reasons studies were downgraded to poor quality was due to ‘assessment of outcomes’ (self-reported measures of dietary intake) and ‘inadequacy of follow-up’ (i.e., high attrition or no comparison between those completed and lost to follow-up), additionally studies did not control for pre-specified demographics or other factors (known confounders) between groups. The one cross-sectional study included in the review was rated as ‘fair’ quality and scored 6 out of a possible 10 stars. The study received at least one star rating for all items within the quality assessment tool with the exception of ‘sample size calculations’ and ‘non-response’ characteristics (due to not being reported).

### 3.3. Primary Outcomes

A meta-analysis was unable to be performed due to heterogeneity in both the definitions and measurement of engagement and diet outcomes. Table 1 provides an overview of all associations (including direction and strength) and Table 4 reports a synthesis of the associations included in the vote count. Overall, none of the included studies reported significant negative associations between measures of engagement and dietary outcomes. The vote count included 14 reports of an association between a measure of engagement and dietary intake across 7 studies (Table 4). Of the 14 associations, 12 were of usage [17,36,38,39,40] and two were of subjective experience [37,41]. Of the 12 usage measures, nine were of logins [17,36,38,39,40], and one each was of ‘time on site’ [36], ‘composite usage’ [34] and ‘activities completed’ [40]. Five (36%) associations were significant and positive and the remaining nine were inconclusive (Table 4). The five positive associations all assessed associations between ‘usage’ measures of engagement and dietary outcomes [17,36,38,39,40].

#### 3.3.1. Usage

##### Logins

Five studies reported associations between logins and dietary intake outcomes (Table 4). From this, logins were found to be consistently and positively associated with fruit intake only (2 of 2 associations) [37,38]. Associations between logins and ‘fruit and vegetable’ intake (1 of 2 associations were significant) and ‘vegetable’ intake (1 of 3 associations were significant) were mixed. Associations between logins and other dietary intake outcomes were non–significant (*n* = 2). Both studies that examined an association between fruit intake and logins were cohort designs [37,38]. The study conducted by Moore et al. of a web-based nutrition education program found that fruit intake at 12 months follow-up was greater among those with more logins (data on estimate not available; *p* = 0.03) [37]. The study conducted by Rodgers et al. which involved participants uploading a photograph of each meal to a website and receiving motivational text messages found that logins (posted photos) were positively associated with intake of fruit (Estimate = 0.017, SE = 0.008, *p* < 0.05) but not for vegetables or calories from sugar-sweetened drinks at a 3-week follow-up [38].

##### Time on Site

One study reported an association for ‘time on site’ and dietary intake [36]. The study reported 20 associations between ‘time’ and change in fruit-and-vegetable intake (Table 1) [36], with ‘time on website’ consistently associated with improvements in fruit-and-vegetable intake (*n* = 3 of 3 associations were significant) compared to time on a specific website feature (*n* = 3 of 17 associations were significant). Of the 20 reported associations, ‘time on site using an adjusted model’ was selected as the measure to represent this paper for inclusion in the vote count (Table 4). From this, time was found to be associated positively with fruit-and-vegetable intake (Estimate = 0.74; SD = 0.19; *p* = 0.001).

##### Composite Usage Measures

One study reported an association between measures of composite usage and dietary intake [42]. Using ordinary least squares regression analyses, the study reported a positive association between the ‘breadth’ (i.e., total website activity e.g., sum of time on website, page visits and logins) with which participants were engaged in a website and fruit-and-vegetable intake (Coefficients not presented in original study; *p <* 0.001). However, no statistically significant association was found when assessing the ‘depth’ (how deeply users engaged e.g., access to special website features and time relative to logins) of website engagement and fruit-and-vegetable intake (Coefficients not presented in original study; *p* = 0.83) [42].

##### Activities Completed

One study reported an association of ‘activities completed’ and dietary intake [40]. The study found that frequency of recording vegetable intake was associated with improvements in vegetable intake, using a ‘standard’ mobile app (*r* = 0.49; *p* = 0.02) but not when using a ‘gamified version’ of the app (*r* = 0.35; *n* = 24; *p* = 0.09) [40].

#### 3.3.2. Subjective Experience 

Two studies reported associations for subjective experience and dietary intake, the findings of which were inconclusive [37,41]. Both studies used surveys to assess the users subjective experience with the DHI and changes in fruit-and-vegetable intake from baseline to one month [37,41]. The surveys used across the two studies varied, with limited details provided on the length, the validity, the reliability of the tool, or other psychometric properties of the tools used. For example, Kothe et al. [41] used a questionnaire to assess user satisfaction, ease-of-use, and interest with the DHI. Lippke et al. [37] used a ‘validated task engagement scale’ and required participants to rate a series of statements (e.g., “I was so immersed, I completely forgot everything else around me”), which was then constructed into an ‘engagement survey score’. The first study, by Kothe et al., found that individuals receiving an email intervention reported mixed findings (Pearson correlation range = 0.002 to 0.163; *p*-value range = ‘not significant’ to <0.05) when assessing measures of subjective experience such as user satisfaction, ease of use and interest with the DHI and fruit-and-vegetable intake [41]. The second study, by Lippke et al., did not find a positive linear relationship between an ‘engagement survey score’ and fruit-and-vegetable intake (Pearson correlation = 0.01; ‘not significant’) after use of an action planning website targeting fruit-and-vegetable intake [37]. 

## 4. Discussion

The aim of the review was to describe the association between engagement with DHIs and improvements in dietary intake. The review included seven studies reported in nine articles. There was considerable heterogeneity in the reporting of both engagement and diet outcomes and the quality of evidence, for a majority of the studies, was considered poor. Overall, the review found mixed evidence supporting an association between DHI usage and dietary intake (five of twelve usage associations were positive, seven were inconclusive). The review found no evidence regarding an association with subjective experience (zero of two associations were inconclusive). Overall, the few studies included in the review, heterogeneity in outcomes and poor quality of evidence limit our ability to draw meaningful conclusions and clearly indicate a need for future research in this area given the wide-held belief that engagement is associated with effectiveness of DHIs.

The mixed findings of this review are in contrast with two other reviews examining the relationship between DHI engagement and other health behaviours [8,20]. The first review, conducted by Donkin et al., reported a consistent positive association between DHI usage and physical health outcomes including fruit-and-vegetable intake, physical activity, weight management and reductions in smoking and smokeless tobacco use [8]. Similarly, a systematic review published in 2021 by Mclaughlin et al. that examined the association between physical activity and DHI engagement found a weak but positive relationship between both DHI usage and physical activity (0.08 [95% CI 0.01, 0.14], n = 11 studies) and a consistent positive association between subjective experience and physical activity (n = 3 studies) [20].

Like the review by Mclaughlin, we found that logins were the most common engagement outcome reported by included studies. However, unlike the Mclaughlin review, logins were not consistently positively associated with measures of dietary intake and were only significantly associated with intake of fruit, but not ‘vegetables’, ‘calories from sugar-sweetened drinks’, and ‘intake from other food groups’. The findings may provide some early evidence to suggest that the relationship between DHI usage (including that assessed via logins) and behaviour change may differ depending on the target behaviour, at least for dietary outcomes. Additionally, these findings may reflect the effectiveness of DHIs and dietary interventions more broadly, where there is a smaller impact on vegetables compared to fruit [44]. More research is required to examine any differences in the relationship between DHI engagement and health behaviours (physical activity vs diet) and across different dietary habits. Alternatively, the differences in the reported associations of logins between this review and that of Mclaughlin et al. may be due to two of the five included studies reporting multiple tests of association of logins and a range of dietary intake outcomes. Multiple testing of dietary outcomes which may have been targeted by the DHI may produce spurious findings [38,39]. Future research that limits multiple-hypothesis testing (due to multiple dietary outcomes) or more intentionally pre-specifies the primary dietary outcomes to align with the target of the intervention and considers consolidated dietary-intake measures (such as diet quality indexes) in their assessment of association may be warranted. 

Consistent with other reviews conceptualising DHI engagement [15] we identified only two studies examining measures of subjective experience. Measures of subjective experience can provide important information on why users engage with DHIs [29] and are hypothesized to predict greater DHI effectiveness [15]. Since publication of the included studies, validated instruments to assess subjective experience have been developed [26]. As such, future research should be undertaken using consistent measures of subjective experience to enable valid and reliable comparisons between studies. This review also identified no studies that used physiological measures of engagement, such as eye tracking, which can often capture which characteristics of the DHI attract attention [29]. For example, eye tracking may capture ‘passive engagement’ or ‘lurking’ whereby users may view, read, and benefit from content posted in forums or on social media posts but do not actively interact with the DHI [29]. These measures also provide objective data on user’s attention, interest, and affect within the DHI and could provide insight into the differential level of engagement for different dietary targets. The lack of studies using physiological measures of engagement is largely consistent with findings of other research in the DHI engagement field [15,29]. As such, future research using broader engagement measures are required to better understand the relationship between DHI engagement and improvements in dietary intake.

A key strength of this review was the comprehensive search strategy, which included screening of 10,653 citations, utilizing published search filters and manual searching in relevant journals and of grey literature. Another strength was that it included measures of subjective experience, a key engagement outcome often overlooked in previous research and suggested to be an important predictor of DHI effectiveness [15]. Despite this, the review should be interpreted in the context of its limitations. First, the heterogeneity of engagement outcomes in studies precluded meta-analyses, an issue reported in previous reviews of engagement [8,24,45]. As such we provide a narrative synthesis and rely on methods such as vote counting in order to synthesize study findings. Second, the inclusion of additional databases in the search may have resulted in additional included studies. Finally, all but two studies were rated as ‘poor quality’, studies were primarily downgraded due to their analysis not being described clearly, which was often a result of the association being included as exploratory or process data rather than a primary or secondary study outcome.

## 5. Conclusions

The current review addresses an important knowledge gap in the engagement literature and is the first to synthesize the association between DHI engagement and dietary intake. The findings suggest there is some evidence supporting an association with usage, however this was inconsistent. No evidence was found regarding an association with subjective experience. Whilst it has been hypothesized that the modest effects of dietary DHIs are due to poor engagement [18], the findings do not yet support this and provide little guidance as to which components of engagement to target to enhance the effectiveness of DHIs. Given the reliance on many public health nutrition strategies on DHIs [30,46], a better understanding of the nature of the relationship is a priority for the field. Specifically, the development and application of consistent and comprehensive measures of the multiple dimensions of engagement is required, and the use of more nuanced, mixed-method, and qualitative approaches may be required to better understand the relationship between DHIs and engagement. In particular, it has been hypothesized that the relationship between engagement and behavior change is unlikely to be linear [37] and that greater engagement may not necessarily yield greater effects on behavior change. Furthermore, we must better understand how relationships are altered by other important contextual factors, including the nature of the DHI, the complexity of the target behavior, and other influences. Ultimately knowing when, and what type of engagement is most important for which behavioral targets, and in what context, will optimize the effects of DHIs for community nutrition improvement.

## Figures and Tables

**Table 1:** Characteristics of included studies.

Author and Study Characteristics	Description of Digital Health Intervention	Engagement Outcome/s	Dietary Outcome/s	Association ^(b)^	Direction of Association ^(c)^	Favorable ^(d)^
**Author:** Alexander 2010; (also reported by Couper 2010) **Design ^(a)^:** cohort **N** = 2513 (baseline) **Age:** 46.3 (SD 10.8) **Female** = 69%	**Type:** Website **Description:** Three-arm website intervention, all arms included access to a basic website with varying levels of tailoring. Arm 1 was the basic site, Arm 2 was a tailored website, Arm 3 was a tailored website with motivational interviewing via email. Web sessions were delivered at 1, 3, 13, and 15 weeks. Participants received $2 incentive prior to entering study & $20 for completing study **Intervention target:** Adults (21–65 years) with no existing health conditions who were registered in a health care system database **Total duration of DHI:** 12 months *	Logins dichotomized into high (>14 logins); medium (7–13 logins); and low (<7 logins) groups	Change in mean servings of fruit and vegetables from baseline to 12 months using a 16-item valid FFQ	low (mean change 2.1); medium (mean change 2.5); high (mean change 3.1) *p* < 0.001	+	√
		Breadth-the sum of four measures, standardized by dividing by their standard deviation. including: total session accesses, unique session access, total special feature accesses, total time online in minutes	Change in mean servings of fruit and vegetables from baseline to 12 months usinga 16-item valid FFQ	Coefficients not presented; *p* < 0.001	+	√
			Change in mean servings of fruit and vegetables from baseline to 12 months usinga 2-item valid FFQ	Coefficients not presented; *p* < 0.001	+	√
		Depth-sum of average total special features sessions; standardized minutes spent online subtracted by twice total number (standardized) of unique sessions	Change in mean servings of fruit and vegetables from baseline to 12 months using a 16-item valid FFQ	Coefficients not presented; *p* = 0.83	0	N/A
			Change in mean servings of fruit and vegetables from baseline to 12 months using a 2-item valid FFQ	Coefficients not presented; *p* = 0.92	0	N/A
**Author:** Buller 2008; (also reported by Woodall 2007) **Design ^(a)^:** cohort **N** = 380 (baseline) **Age ^(c)^:** <29 years = 35% **Female** = 88%	**Type:** Website **Description:** Fruit and vegetable nutrition education website (password protected), participants were contacted by research team to log onto website once each month, every 2 months participants received ‘small gift’ as a reminder to visit website, routine email notifications were sent announcing new content. **Intervention target:** Adults (>18 years old), English speaking and living in Southwestern USA for at least 6 months. **Total duration of DHI:** 4 months	Time on website (mean minutes)	Change in mean servings of fruit-and-vegetable intake from baseline to 4 months using valid **all day** screener (ranked pre- and post-test)	Unadjusted: R = 0.14, *p* = 0.004	+	√
				Adjusted: Estimate = 0.74, SD = 0.19, t(df = 414) = 3.87, *p* = 0.001	+	√
		Time on website (mean minutes)	Change in mean servings of fruit-and-vegetable intake from baseline to 4 months using **single item** screener (ranked pre- and post-test)	OR (95% CI) 1.010 (1.003, 1.018) per minute of use	+	√
		Time on website features (mean minutes)	Change in mean servings of fruit-and-vegetable intake from baseline to 4 months using valid **all day** screener (ranked pre- and post-test)	17 associations Range of means (SD): 0.009 (0.096) to 13.745 (21.203) Range of Spearman correlation: −0.076 to 0.185 Range of *p* value: 0.0064 to 0.9189 (only 3 significant)	N/A ^(e)^	N/A
		Number of logins within 5 days of an email	Change in mean servings of fruit-and-vegetable intake from baseline to 4 months using valid FFQ	coefficient = 0.14, *p* = 0.049	+	√
		Proportion of logins after email	Change in mean servings of fruit-and-vegetable intake from baseline to 4 months using valid FFQ	coefficient = 0.11, *p* = 0.12	0	N/A
**Author:** Kothe 2014 **Design ^(a)^:** cohort **N** = 217 (baseline) **Age:** 18.92 (SD 1.37) **Female** = 77.3%	**Type:** Email intervention **Description:** Email intervention with two levels of message frequency. Participants in high frequency intervention arm received emails daily (27 emails in total) and those in low frequency arm received emails every 3 days (9 emails in total). Course credit was provided for participating students **Intervention target:** Adults (>18 years) who were an undergraduate psychology student at an Australian University **Total duration of DHI:** 30 days	Subjective experience using Likert scale: Interest	Change in fruit-and-vegetable intake scores (servings/day) from baseline to 30 days using self-report e.g., *“How many servings of fruit did you eat yesterday?”*	Correlation = 0.163, *p* < 0.05	+	√
		- Credibility		Correlation = 0.002, *p* = ‘not significant’	0	N/A
		- Logical		Correlation = −0.034, *p* = ‘not significant’	0	N/A
		- Easy to understand		Correlation = 0.021, *p* = ‘not significant’	0	N/A
		- Relevant		Correlation = 0.102, *p* = ‘not significant’	0	N/A
		- Useful		Correlation = 0.149, *p* < 0.05	+	√
		- Complete		Correlation = 0.146, *p* < 0.05	+	√
		- Too long		Correlation = −0.032, *p* = ‘not significant’	0	N/A
		- Annoying		Correlation = −0.104, *p* = ‘not significant’	0	N/A
		- Too many emails		Correlation = −0.078, *p* = ‘not significant’	0	N/A
		- Confusing		Correlation = 0.067, *p* = ‘not significant’	0	N/A
**Author:** Lippke 2016 **Design of association^(a)^:** cohort **N** = 701 (at association) **Age:** 38.71 **Female** = 84%	**Type:** Website **Description:** One-off action-planning and coping-planning website aimed to improve fruit-and-vegetable intake. As an incentive for study participation, individuals were able to take part in an optional raffle in which they could win attractive gift certificates for an online bookstore **Intervention target:** Adults **Total duration of DHI:** 1 month	Engagement survey score using Likert scale	Change in fruit-and-vegetable intake scores from baseline to one month (servings/day) using valid ‘open answer’ questionnaire e.g., “*how many servings of (a) fruit…and (b) vegetables…do you eat on average per day?*”	Correlation = 0.01, *p* = ‘not significant’,non-linear relationship observed	0	N/A
**Author:** Moore 2008 **Design ^(a)^:** cohort **N** = 181 (at association) **Age:** not reported **Female** = 59% ^#^	**Type:** Website **Description:** Password-protected website on healthy eating, content was posted each Friday with weekly reminder emails sent to participants. Dietary advice was based on the DASH diet (Dietary Approaches to Stop Hypertension). **Intervention target:** Adult employees of a US based infrastructure company **Total duration of DHI:** 12 months	Number of logins	Change in **fruit** servings from baseline to 12 months using valid FFQ	*p* = 0.03	+	√
			Change in **vegetable** servings from baseline to 12 months using valid FFQ	*p* = ‘not significant’	0	N/A
			Change in **grains** servings from baseline to 12 months using valid FFQ	*p* = ‘not significant’	0	N/A
			Change in **dairy** servings from baseline to 12 months using valid FFQ	*p* = ‘not significant’	0	N/A
			Change in **meat & fish** servings from baseline to 12 months using valid FFQ	*p* = ‘not significant’	0	N/A
			Change in **nut & beans** servings from baseline to 12 months using valid FFQ	*p* = ‘not significant’	0	N/A
			Change in **added fats** servings from baseline to 12 months using valid FFQ	*p* = ‘not significant’	0	N/A
			Change in **sweets** servings from baseline to 12 months using valid FFQ	*p* = ‘not significant’	0	N/A
**Author:** Nour 2019 Design ^(a)^: Cross-sectional **N** = 97 (baseline) **Age:** 24.8 (SD 3.4) **Female** = 60%	**Type** Standard App OR gamified app +/- Facebook **Description:** Standard app of goal setting and self-monitoring with feedback on vegetable intake, Gamified app included rewards as incentivization. Facebook included cooking videos addressing known barriers shared by a dietician daily. **Intervention target:** Adults 18–30 years, who owned a smartphone and lived in New South Wales, Australia **Total duration of DHI:** 4 weeks	Total days of app engagement via recorded logins in *standard app*	Change in vegetable intake (servings/ day) from baseline to 4 weeks using valid short questionnaires	*r* = 1; *n* = 23; *p* < 0.00001	+	√
		Total days of app engagement via recorded logins in gamified app		*r* = 0.64; *n* = 24; *p* = 0.001	+	√
		Frequency of recording vegetable intake via app analytics in standard app		*r* = 0.49; *n* = 23; *p* = 0.02	+	√
		Frequency of recording vegetable intake via app analytics in gamified app		*r* = 0.35; *n* = 24; *p* = 0.09	0	N/A
**Author:** Rodgers 2016 Design ^(a)^: cohort **N** = 46 (baseline) **Age:** 18.96 (SD 0.76) **Female** = 100%	**Type:** Website + SMS **Description:** Participants were encouraged to take photos of meals using their mobile phone and upload them to a website (Photobucket) and received 3 x motivational text messages/day at mealtimes to encourage healthy eating. Intervention target: Full time female undergraduate college students (>18 years) **Total duration of DHI:** 3 weeks	Number of photos posted (logins)	Vegetable intake (servings/day) using a valid 2-item FFQ	Estimate = 0.012, SE = 0.008, *p* = ‘not significant’	0	N/A
			Fruit intake (servings/day) using a valid 2-item FFQ	Estimate = 0.017, SE = 0.008, *p* < 0.05	+	√
			Log of calories from sugar-sweetened beverages using a ‘beverage intake questionnaire’	Estimate = 0.007, SE = 0.009, *p* = ‘not significant’	0	N/A

**^(a)^** Studies were considered to be ‘cohort’ in design if the association was between engagement and change in dietary intake over time; **^(b)^** All available data relating to ‘association’ is presented, including odds ratios (ORs) or regression coefficients or estimates; 95% confidence intervals (CI) or standard deviations (SD) or standard errors (SE) and; *p*-values are presented; **^(c)^** Statistically significant associations denoted as ‘+’, ‘0’, ‘−’. Significant positive linear associations between DHI engagement and effectiveness on dietary intake were denoted by a plus sign (+). Significant negative linear associations between DHI engagement and effectiveness on dietary intake were denoted by a minus sign (−). Non-significant associations were denoted as ‘0′; **^(d)^** Favourable outcomes were denoted with a tick (√) if they were significant and supported the hypothesis that higher engagement is associated with improvements in dietary intake. Unfavourable outcomes were denoted with a cross (***X***) if they were significant and rejected the supported hypothesis. Outcomes marked N/A showed no significant association.

**Table 2:** Results of the critical appraisal of the included studies using the Newcastle—Ottawa Scale ^(a)^ for Cohort studies.

	Selection				Comparability	Outcome			Quality
Study	Representativeness of the Exposed Cohort	Selection of Non-Exposed Cohort	Ascertainment of Exposure	Outcome of Interest	Cohort Statistical Analysis	Assessment of Outcome	Length of Follow Up	Adequacy of Follow Up	
Alexander (2010) & Couper (2010)	★	★	★	★	-	-	★	★	Good
Buller (2008) & Woodall (2007)	★	★	★	-	★	-	★	-	Poor
Kothe (2014)	-	★	-	★	-	-	★	-	Poor
Lippke (2016)	★	★	-	★	★	-	-	-	Poor
Moore (2008)	★	★	★	★	-	-	★	-	Poor
Rodgers (2016)	★	★	★	★	★	-	-	-	Poor

^(a)^ Newcastle-Ottawa scales scoring: Good quality were those that scored 3–4 stars in selection domain AND 1–2 stars in comparability domain AND 2–3 stars in outcome/exposure domain; Fair quality studies were those that scored 2 stars in selection domain AND 1–2 stars in comparability domain AND 2–3 stars in outcome/exposure domain; Poor quality studies were those that scored, 0–1 stars in selection domain OR 0 stars in comparability domain OR 0–1 stars in outcome/exposure domain.

**Table 3:** Results of the critical appraisal of the included studies using the Newcastle—Ottawa Scale ^(a)^ for cross-sectional studies.

	Selection				Comparability	Outcome		Quality
**Study**	Representativeness of the Sample	Sample Size	Non Respondent	Ascertainment of Exposure	Statistical Analysis Design Features	Assessment of Outcome	Statistical Test	
Nour (2019)	★	-	-	★	★ ★	★	★	Fair

^(a)^ Newcastle-Ottawa scales scoring: Good quality: 3–5 stars in selection domain AND 1–2 stars in comparability domain AND 2–3 stars in outcome domain; Fair quality: 2 stars in selection domain AND 1 or 2 stars in comparability domain AND 2 or 3 stars in outcome/exposure domain; Poor quality: 0–1 stars in selection domain OR 0 stars in comparability domain OR 0–1 stars in outcome/exposure domain.

**Table 4:** Summary of associations included in the vote-count.

Study	Diet Measure	Engagement Measure ^(a)^	Analysis	Association	Direction ^(b)^	Favorable ^(c)^
Alexander 2010 & Couper 2010 [17,42]	fruit and vegetables	logins	Kruskal—Wallis test	*p* < 0.001	+	√
	fruit and vegetables	composite usage measure	ordinary least squares regression	2 associations: adjusted model *p* < 0.001; adjusted model *p* = 0.83	0	N/A
Buller 2008 & Woodall 2007 [36,43]	fruit and vegetables	time on website	unclear	adjusted model estimate = 0.74, SD = 0.19 *p* = 0.001	+	√
	fruit and vegetables	logins	non-parametric spearman partial correlation	2 associations: coefficient = 0.14, *p* = 0.049; coefficient = 0.11, *p* = 0.12	0	N/A
Kothe 2014 [41]	fruit and vegetables	subjective experience	Pearson correlation	11 associations: Pearson correlation (range) = 0.002 to 0.163 *p value* (range) = ‘not significant’ to *p* < 0.05	0	N/A
Lippke 2016 [37]	fruit and vegetables	subjective experience	Pearson correlation	Pearson correlation = 0.01, *p* = ‘not significant’	0	N/A
Moore 2008 [38]	fruit	logins	unclear	*p* = 0.03	+	√
	vegetables	logins	unclear	*p* = ‘not significant’	0	N/A
	other food groups	logins	unclear	*p* = ‘not significant’	0	N/A
Nour 2019 [40]	vegetables	logins	spearman correlation	2 associations: r = 1; n = 23; *p* < 0.00001. r = 0.64; n = 24; *p* = 0.001	+	√
	vegetables	activities completed	spearman correlation	2 associations: r = 0.49; n = 23; *p* = 0.02. r = 0.35; n = 24; *p* = 0.09	0	N/A
Rodgers 2016 [39]	vegetables	logins	mixed effects modelling	estimate = 0.012, SE = 0.008, *p* = ‘not significant’	0	N/A
	fruit	logins	mixed effects modelling	estimate = 0.017, SE = 0.008, *p* < 0.05	+	√
	calories from sugar-sweetened drinks	logins	mixed effects modelling	estimate = 0.007, SE = 0.009, *p* = ‘not significant’	0	N/A

^(a)^ Studies were aggregated under the following standardized engagement variable names: (i)‘logins’, (ii) ‘time’, (iii) ‘composite usage measure’, (iv) ‘activities completed’, or (v) ‘subjective experience’. ^(b)^ A single association was selected for each study based on hierarchical criteria, see methods section. Statistically significant associations denoted as ‘+’, ‘0’, ‘−’. Positive linear associations between DHI engagement and effectiveness on dietary intake were denoted by a plus sign (+). Negative linear associations between DHI engagement and effectiveness on dietary intake were denoted by a minus sign (−). Non-significant associations or studies with inconclusive or mixed associations were denoted by (0) regardless of direction. ^(c)^ Favorable outcomes were denoted with a tick (√) if they were significantly and positively associated. Outcomes marked N/A showed no significant association.

## Data Availability

Data is contained within the article or are available from the included studies that have been referenced throughout.

## Supplementary Materials

The following are available online at https://www.mdpi.com/article/10.3390/nu13093281/s1, Supplementary File S1: Search Strategy; Supplementary File S2: PRISMA flow diagram, Supplementary File S3: Newcastle—Ottawa Scale.
