# Supplementary material for: Associations between Digital Health Intervention Engagement and Dietary Intake: A Systematic Review
Source: Nutrients. 2021 Sep 20;13(9):3281. doi: 10.3390/nu13093281 (PMC8470016; doi:10.3390/nu13093281)
Supplement: Supplementary file 1 [file nutrients-13-03281-s001.zip › Supplementary File 1 - Search Strategy.pdf]

Supplementary File 1 – Search Strategy

Engagement and health behaviors

Matthew McLaughlin and Tessa Delaney

Searches conducted: 4/9/18 and 5/9/18 AND then updated 5/12/2019.

Database	Records identified	Records after de-duplication
Academic Search Ultimate	2287	710
CENTRAL	2844	1882
CINAHL	965	321
Embase	2621	768
Medline	1805	414
PsycINFO	897	146
Scopus	1773	691
**TOTAL**	**13,192**	**4932**

Database(s): **Ovid MEDLINE(R) and Epub Ahead of Print, In-Process & Other Non-Indexed Citations and Daily**
Search Strategy:

#	Searches
1	exercise/ or gymnastics/ or physical conditioning, human/ or running/ or swimming/ or walking/
2	Motor Activity/
3	(physical education and training).mp.
4	physical fitness/ or cardiorespiratory fitness/
5	life style/ or healthy lifestyle/ or healthy aging/ or healthy diet/ or sedentary lifestyle/
6	exp sports/
7	physical* inactiv*.ti,ab,kw.
8	physical* activ*.ti,ab,kw.
9	sedentary.ti,ab,kw.
10	Exercis*.ti,ab,kw.
11	Sport*.ti,ab,kw.
12	(("lifestyle*" or life style*) adj5 activ*).ti,ab,kw.
13	(fitness adj (class* or regime* or program*)).mp.
14	1 or 2 or 3 or 4 or 5 or 6 or 7 or 8 or 9 or 10 or 11 or 12 or 13
15	exp Diet/
16	nutrition*.ti,ab,kw.
17	Healthy eating.ti,ab,kw.
18	Child nutrition sciences/
19	fruit*.ti,ab,kw.
20	Fruit/
21	Vegetable*.ti,ab,kw.
22	Vegetables/
23	Canteen*.ti,ab,kw.
24	Food services/ or food service*.ti,ab,kw.
25	Menu planning/ or menu*.ti,ab,kw.
26	calorie*.ti,ab,kw.
27	Energy intake/
28	energy density.ti,ab,kw.
29	eating/
30	Feeding Behavior/ or feeding behavio*.ti,ab,kw.
31	dietary intake.ti,ab,kw.

32	food habits.ti,ab,kw.
33	food/
34	Carbonated Beverages/ or soft drink*.ti,ab,kw.
35	soda.ti,ab,kw.
36	sweetened drink*.ti,ab,kw.
37	Dietary Fats, Unsaturated/ or Dietary Fats/
38	confectionar*.ti,ab,kw.
39	(school adj (lunch* or meal*)).ti,ab,kw.
40	((feeding or food or nutrition*) adj program*).ti,ab,kw.
41	cafeteria*.ti,ab,kw.
42	(food adj2 (purchas* or select*)).ti,ab,kw.
43	15 or 16 or 17 or 18 or 19 or 20 or 21 or 22 or 23 or 24 or 25 or 26 or 27 or 28 or 29 or 30 or 31 or 32 or 33 or 34 or 35 or 36 or 37 or 38 or 39 or 40 or 41 or 42
44	System* usability scale.ti,ab,kw.
45	((intervention or app or e-mail or email or m-health or e-health or digital health or online or web*) adj5 (adopt* or uptake or retention or maintenance or participat* or engag* or usage attrition or usage or adher*)).ti,ab,kw.
46	(process adj (metric* or evaluation)).ti,ab,kw.
47	login*.ti,ab,kw.
48	(page* adj3 view*).ti,ab,kw.
49	((module* or session*) adj complet*).ti,ab,kw.
50	(visit* adj3 website*).ti,ab,kw.
51	(time adj3 online).ti,ab,kw.
52	(user adj3 (engag* or experience* or enjoyment or attention or interest or affect or immersion or involvement)).ti,ab,kw.
53	usability.ti,ab,kw.
54	44 or 45 or 46 or 47 or 48 or 49 or 50 or 51 or 52 or 53
55	Smartphone/ or Cell Phone/ or mobile device*.ti,ab,kw. or Mobile Applications/
56	Virtual reality/ or (virtual adj reality).ti,ab,kw.
57	Online Systems/ or online.ti,ab,kw.
58	Internet.ti,ab,kw.
59	(web adj based).ti,ab,kw.
60	(world wide web or WWW or website*).ti,ab,kw.
61	Electronic Mail/ or (e-mail* or email*).ti,ab,kw.

62	((mobile or cellular or cell or smart) adj (phone* or telephone*)).ti,ab,kw.
63	(e-health or ehealth or electronic health).ti,ab,kw.
64	(m-health or mhealth or mobile health).ti,ab,kw.
65	((digital health or digital) adj3 intervention*).ti,ab,kw.
66	(interactive adj ((health adj communicat*) or video* or technolog* or multimedia)).ti,ab,kw.
67	((chat adj room*) or chatroom*).ti,ab,kw.
68	(phone adj app*).ti,ab,kw.
69	User-Computer Interface/ or human computer interaction*.ti,ab,kw.
70	55 or 56 or 57 or 58 or 59 or 60 or 61 or 62 or 63 or 64 or 65 or 66 or 67 or 68 or 69
71	14 or 43
72	54 and 70 and 71
73	animals/ not (humans/ and animals/)
74	72 not 73
**75**	**limit 74 to english language**

Database(s): **Embase** 1947 to present
Search Strategy:

#	Searches
1	exercise/
2	physical education/
3	running/
4	swimming/
5	walking/
6	motor activity/
7	fitness/
8	cardiorespiratory fitness/
9	lifestyle/ or healthy lifestyle/ or sedentary lifestyle/
10	healthy aging/
11	healthy diet/
12	exp sport/
13	physical activity/ or physical inactivity/
14	(physical* inactiv* or physical* activ*).ti,ab,kw.
15	sedentary.ti,ab,kw.
16	Exercis*.ti,ab,kw.
17	Sport*.ti,ab,kw.
18	(("lifestyle*" or life style*) adj5 activ*).ti,ab,kw.
19	(fitness adj (class* or regime* or program*)).mp.
20	(gymnastic* or physical conditioning).ti,ab,kw.
21	1 or 2 or 3 or 4 or 5 or 6 or 7 or 8 or 9 or 10 or 11 or 12 or 13 or 14 or 15 or 16 or 17 or 18 or 19 or 20
22	exp diet/
23	nutrition*.ti,ab,kw.
24	Healthy eating.ti,ab,kw.
25	Child nutrition sciences.ti,ab,kw.
26	fruit*.ti,ab,kw.
27	fruit/
28	Vegetable*.ti,ab,kw.
29	vegetable/
30	Canteen*.ti,ab,kw.
31	catering service/

32	food service*.ti,ab,kw.
33	menu*.ti,ab,kw.
34	calorie*.ti,ab,kw.
35	caloric intake/
36	energy density.ti,ab,kw.
37	eating/
38	Feeding Behavior/ or feeding behavio*.ti,ab,kw.
39	dietary intake.ti,ab,kw.
40	food habits.ti,ab,kw.
41	food/
42	Carbonated Beverage/ or soft drink*.ti,ab,kw.
43	soda.ti,ab,kw.
44	sweetened drink*.ti,ab,kw.
45	fat intake/ or dietary intake/
46	confectionar*.ti,ab,kw.
47	(school adj (lunch* or meal*)).ti,ab,kw.
48	((feeding or food or nutrition*) adj program*).ti,ab,kw.
49	cafeteria*.ti,ab,kw.
50	(food adj2 (purchas* or select*)).ti,ab,kw.
51	22 or 23 or 24 or 25 or 26 or 27 or 28 or 29 or 30 or 31 or 32 or 33 or 34 or 35 or 36 or 37 or 38 or 39 or 40 or 41 or 42 or 43 or 44 or 45 or 46 or 47 or 48 or 49 or 50
52	System* usability scale.ti,ab,kw.
53	((intervention or app or e-mail or email or m-health or e-health or digital health or online or web*) adj5 (adopt* or uptake or retention or maintenance or participat* or engag* or usage attrition or usage or adher*)).ti,ab,kw.
54	(process adj (metric* or evaluation)).ti,ab,kw.
55	login*.ti,ab,kw.
56	(page* adj3 view*).ti,ab,kw.
57	((module* or session*) adj complet*).ti,ab,kw.
58	(visit* adj3 website*).ti,ab,kw.
59	(time adj3 online).ti,ab,kw.
60	(user adj3 (engag* or experience* or enjoyment or attention or interest or affect or immersion or involvement)).ti,ab,kw.
61	usability.ti,ab,kw.

62	52 or 53 or 54 or 55 or 56 or 57 or 58 or 59 or 60 or 61
63	smartphone/ or mobile phone/
64	mobile application/
65	mobile device*.ti,ab,kw.
66	Virtual reality/ or (virtual adj reality).ti,ab,kw.
67	Online System/ or online.ti,ab,kw.
68	Internet/ or Internet.ti,ab,kw.
69	(web adj based).ti,ab,kw.
70	(world wide web or WWW or website*).ti,ab,kw.
71	e-Mail/ or (e-mail* or email* or electronic mail).ti,ab,kw.
72	((mobile or cellular or cell or smart) adj (phone* or telephone*)).ti,ab,kw.
73	(e-health or ehealth or electronic health).ti,ab,kw.
74	(m-health or mhealth or mobile health).ti,ab,kw.
75	((digital health or digital) adj3 intervention*).ti,ab,kw.
76	(interactive adj ((health adj communicat*) or video* or technolog* or multimedia)).ti,ab,kw.
77	((chat adj room*) or chatroom*).ti,ab,kw.
78	(phone adj app*).ti,ab,kw.
79	Computer Interface/ or human computer interaction*.ti,ab,kw.
80	63 or 64 or 65 or 66 or 67 or 68 or 69 or 70 or 71 or 72 or 73 or 74 or 75 or 76 or 77 or 78 or 79
81	21 or 51
82	62 and 80 and 81
83	animal/ not (human/ and animal/)
84	82 not 83
85	limit 84 to english language

Database(s): **PsycINFO**
Search Strategy:

#	Searches
1	exercise/ or physical activity/ or physical fitness/
2	exp sports/
3	lifestyle/ or active living/ or lifestyle changes/
4	(physical education and training).mp.
5	(gymnastics or physical conditioning or running or walking).ti,ab,id.
6	motor activity.ti,ab,id.
7	(physical education and training).ti,ab,id.
8	(physical activ* or physical* inactiv*).ti,ab,id.
9	(sedentary or exercis* or sport* or healthy diet or healthy ag?ing).ti,ab,id.
10	(("lifestyle*" or life style*) adj5 activ*).ti,ab,id.
11	(fitness adj (class* or regime* or program*)).ti,ab,id.
12	1 or 2 or 3 or 4 or 5 or 6 or 7 or 8 or 9 or 10 or 11
13	diets/
14	nutrition*.ti,ab,id.
15	Healthy eating.ti,ab,id.
16	(fruit* or vegetable*).ti,ab,id.
17	(canteen* or food service* or menu* or calorie*).ti,ab,id.
18	Food Intake/
19	Eating Behavior/
20	"beverages (nonalcoholic)"/
21	FOOD/
22	(energy intake or energy density or feeding behavio* or dietary intake or food habits or carbonated beverage* or soft drink* or soda or sweetened drink* or dietary fat* or confectionar*).ti,ab,id.
23	(school adj (lunch* or meal*)).ti,ab,id.
24	((feeding or food or nutrition*) adj program*).ti,ab,id.
25	cafeteria*.ti,ab,id.
26	(food adj2 (purchas* or select*)).ti,ab,id.
27	13 or 14 or 15 or 16 or 17 or 18 or 19 or 20 or 21 or 22 or 23 or 24 or 25 or 26
28	System* usability scale.ti,ab,id.
29	((intervention or app or e-mail or email or m-health or e-health or digital health or online or web*) adj5 (adopt* or uptake or retention or maintenance or participat* or engag* or usage attrition or usage or adher*)).ti,ab,id.

30	(process adj (metric* or evaluation)).ti,ab,id.
31	login*.ti,ab,id.
32	(page* adj3 view*).ti,ab,id.
33	((module* or session*) adj complet*).ti,ab,id.
34	(visit* adj3 website*).ti,ab,id.
35	(time adj3 online).ti,ab,id.
36	(user adj3 (engag* or experience* or enjoyment or attention or interest or affect or immersion or involvement)).ti,ab,id.
37	usability.ti,ab,id.
38	28 or 29 or 30 or 31 or 32 or 33 or 34 or 35 or 36 or 37
39	exp Cellular Phones/ or exp Mobile Devices/
40	mobile device*.ti,ab,id.
41	Virtual reality/ or (virtual adj reality).ti,ab,id.
42	online.ti,ab,id.
43	Internet/ or Internet.ti,ab,id.
44	(web adj based).ti,ab,id.
45	(world wide web or WWW or website*).ti,ab,id.
46	Computer Mediated Communication/ or (e-mail* or email* or electronic mail).ti,ab,id.
47	((mobile or cellular or cell or smart) adj (phone* or telephone*)).ti,ab,id.
48	(e-health or ehealth or electronic health).ti,ab,id.
49	(m-health or mhealth or mobile health).ti,ab,id.
50	((digital health or digital) adj3 intervention*).ti,ab,id.
51	(interactive adj ((health adj communicat*) or video* or technolog* or multimedia)).ti,ab,id.
52	((chat adj room*) or chatroom*).ti,ab,id.
53	(phone adj app*).ti,ab,id.
54	Human Computer Interface/ or human computer interaction*.ti,ab,id.
55	39 or 40 or 41 or 42 or 43 or 44 or 45 or 46 or 47 or 48 or 49 or 50 or 51 or 52 or 53 or 54
56	12 or 27
57	38 and 55 and 56
58	limit 57 to (human and english language)

CINAHL

#	Query	Limiters/Expanders	Last Run Via
S1	(MH "Exercise")	Search modes - Boolean/Phrase	Interface - EBSCOhost Research Databases Search Screen - Advanced Search Database - CINAHL Complete
S2	(MH "Gymnastics")	Search modes - Boolean/Phrase	Interface - EBSCOhost Research Databases Search Screen - Advanced Search Database - CINAHL Complete
S3	(MH "Running")	Search modes - Boolean/Phrase	Interface - EBSCOhost Research Databases Search Screen - Advanced Search Database - CINAHL Complete
S4	(MH "Swimming")	Search modes - Boolean/Phrase	Interface - EBSCOhost Research Databases Search Screen - Advanced Search Database - CINAHL Complete
S5	(MH "Walking")	Search modes - Boolean/Phrase	Interface - EBSCOhost Research Databases Search Screen - Advanced Search Database - CINAHL Complete
S6	(MH "Motor Activity")	Search modes - Boolean/Phrase	Interface - EBSCOhost Research Databases Search Screen - Advanced Search Database - CINAHL Complete
S7	(MH "Physical Education and Training")	Search modes - Boolean/Phrase	Interface - EBSCOhost Research Databases Search Screen - Advanced Search Database - CINAHL Complete
S8	(MH "Physical Fitness")	Search modes - Boolean/Phrase	Interface - EBSCOhost Research Databases Search Screen - Advanced Search Database - CINAHL Complete
S9	(MH "Cardiorespiratory Fitness")	Search modes - Boolean/Phrase	Interface - EBSCOhost Research Databases Search Screen - Advanced Search Database - CINAHL Complete
S10	(MH "Life Style") OR (MH "Life Style, Sedentary")	Search modes - Boolean/Phrase	Interface - EBSCOhost Research Databases Search Screen - Advanced Search Database - CINAHL Complete
S11	"healthy aging"	Search modes - Boolean/Phrase	Interface - EBSCOhost Research Databases Search Screen - Advanced Search Database - CINAHL Complete
S12	"healthy diet"	Search modes - Boolean/Phrase	Interface - EBSCOhost Research Databases Search Screen - Advanced Search Database - CINAHL Complete
S13	(MH "Sports+")	Search modes - Boolean/Phrase	Interface - EBSCOhost Research Databases Search Screen - Advanced Search Database - CINAHL Complete

S14	TI "physical* inactiv*" OR AB "physical* inactiv*"	Search modes - Boolean/Phrase	Interface - EBSCOhost Research Databases<br>Search Screen - Advanced Search<br>Database - CINAHL Complete
S15	TI "physical* activ*" OR AB "physical* activ*"	Search modes - Boolean/Phrase	Interface - EBSCOhost Research Databases<br>Search Screen - Advanced Search<br>Database - CINAHL Complete
S16	TI sedentary OR AB sedentary	Search modes - Boolean/Phrase	Interface - EBSCOhost Research Databases<br>Search Screen - Advanced Search<br>Database - CINAHL Complete
S17	TI Exercis* OR AB Exercis*	Search modes - Boolean/Phrase	Interface - EBSCOhost Research Databases<br>Search Screen - Advanced Search<br>Database - CINAHL Complete
S18	TI Sport* OR AB Sport*	Search modes - Boolean/Phrase	Interface - EBSCOhost Research Databases<br>Search Screen - Advanced Search<br>Database - CINAHL Complete
S19	TI ((("lifestyle*" or life style*) n5 activ*)) OR AB ((("lifestyle*" or life style*) n5 activ*))	Search modes - Boolean/Phrase	Interface - EBSCOhost Research Databases<br>Search Screen - Advanced Search<br>Database - CINAHL Complete
S20	TI ((fitness n1 (class* or regime* or program*))) OR AB ((fitness n1 (class* or regime* or program*)))	Search modes - Boolean/Phrase	Interface - EBSCOhost Research Databases<br>Search Screen - Advanced Search<br>Database - CINAHL Complete
S21	S1 OR S2 OR S3 OR S4 OR S5 OR S6 OR S7 OR S8 OR S9 OR S10 OR S11 OR S12 OR S13 OR S14 OR S15 OR S16 OR S17 OR S18 OR S19 OR S20	Search modes - Boolean/Phrase	Interface - EBSCOhost Research Databases<br>Search Screen - Advanced Search<br>Database - CINAHL Complete
S22	(MH "Diet+")	Search modes - Boolean/Phrase	Interface - EBSCOhost Research Databases<br>Search Screen - Advanced Search<br>Database - CINAHL Complete
S23	(MH "Nutrition") OR "nutrition*"	Search modes - Boolean/Phrase	Interface - EBSCOhost Research Databases<br>Search Screen - Advanced Search<br>Database - CINAHL Complete
S24	TI "Healthy eating" OR AB "Healthy eating"	Search modes - Boolean/Phrase	Interface - EBSCOhost Research Databases<br>Search Screen - Advanced Search<br>Database - CINAHL Complete
S25	"Child nutrition sciences"	Search modes - Boolean/Phrase	Interface - EBSCOhost Research Databases<br>Search Screen - Advanced Search<br>Database - CINAHL Complete

S26	TI fruit* OR AB fruit*	Search modes - Boolean/Phrase	Interface - EBSCOhost Research Databases<br>Search Screen - Advanced Search<br>Database - CINAHL Complete
S27	(MH "Fruit")	Search modes - Boolean/Phrase	Interface - EBSCOhost Research Databases<br>Search Screen - Advanced Search<br>Database - CINAHL Complete
S28	TI Vegetable* OR AB Vegetable*	Search modes - Boolean/Phrase	Interface - EBSCOhost Research Databases<br>Search Screen - Advanced Search<br>Database - CINAHL Complete
S29	(MH "Vegetables")	Search modes - Boolean/Phrase	Interface - EBSCOhost Research Databases<br>Search Screen - Advanced Search<br>Database - CINAHL Complete
S30	TI Canteen* OR AB Canteen*	Search modes - Boolean/Phrase	Interface - EBSCOhost Research Databases<br>Search Screen - Advanced Search<br>Database - CINAHL Complete
S31	(MH "Food Services") OR "food service*"	Search modes - Boolean/Phrase	Interface - EBSCOhost Research Databases<br>Search Screen - Advanced Search<br>Database - CINAHL Complete
S32	(MH "Menu Planning") OR "menu*"	Search modes - Boolean/Phrase	Interface - EBSCOhost Research Databases<br>Search Screen - Advanced Search<br>Database - CINAHL Complete
S33	"calorie*"	Search modes - Boolean/Phrase	Interface - EBSCOhost Research Databases<br>Search Screen - Advanced Search<br>Database - CINAHL Complete
S34	(MH "Energy Intake")	Search modes - Boolean/Phrase	Interface - EBSCOhost Research Databases<br>Search Screen - Advanced Search<br>Database - CINAHL Complete
S35	(MH "Energy Density")	Search modes - Boolean/Phrase	Interface - EBSCOhost Research Databases<br>Search Screen - Advanced Search<br>Database - CINAHL Complete
S36	(MH "Eating")	Search modes - Boolean/Phrase	Interface - EBSCOhost Research Databases<br>Search Screen - Advanced Search<br>Database - CINAHL Complete
S37	(MH "Eating Behavior")	Search modes - Boolean/Phrase	Interface - EBSCOhost Research Databases<br>Search Screen - Advanced Search<br>Database - CINAHL Complete
S38	TI dietary intake OR AB dietary intake	Search modes - Boolean/Phrase	Interface - EBSCOhost Research Databases<br>Search Screen - Advanced Search<br>Database - CINAHL Complete

S39	(MH "Food Habits")	Search modes - Boolean/Phrase	Interface - EBSCOhost Research Databases Search Screen - Advanced Search Database - CINAHL Complete
S40	(MH "Food")	Search modes - Boolean/Phrase	Interface - EBSCOhost Research Databases Search Screen - Advanced Search Database - CINAHL Complete
S41	(MH "Carbonated Beverages")	Search modes - Boolean/Phrase	Interface - EBSCOhost Research Databases Search Screen - Advanced Search Database - CINAHL Complete
S42	TI "soft drink*" OR AB "soft drink*"	Search modes - Boolean/Phrase	Interface - EBSCOhost Research Databases Search Screen - Advanced Search Database - CINAHL Complete
S43	TI soda OR AB soda	Search modes - Boolean/Phrase	Interface - EBSCOhost Research Databases Search Screen - Advanced Search Database - CINAHL Complete
S44	TI "sweetened drink*" OR AB "sweetened drink*"	Search modes - Boolean/Phrase	Interface - EBSCOhost Research Databases Search Screen - Advanced Search Database - CINAHL Complete
S45	(MH "Dietary Fats")	Search modes - Boolean/Phrase	Interface - EBSCOhost Research Databases Search Screen - Advanced Search Database - CINAHL Complete
S46	TI confectionar* OR AB confectionar*	Search modes - Boolean/Phrase	Interface - EBSCOhost Research Databases Search Screen - Advanced Search Database - CINAHL Complete
S47	TI ((school n1 (lunch* or meal*))) OR AB ((school n1 (lunch* or meal*)))	Search modes - Boolean/Phrase	Interface - EBSCOhost Research Databases Search Screen - Advanced Search Database - CINAHL Complete
S48	TI (((feeding or food or nutrition*) n1 program*)) OR AB (((feeding or food or nutrition*) n1 program*))	Search modes - Boolean/Phrase	Interface - EBSCOhost Research Databases Search Screen - Advanced Search Database - CINAHL Complete
S49	TI cafeteria* OR AB cafeteria*	Search modes - Boolean/Phrase	Interface - EBSCOhost Research Databases Search Screen - Advanced Search Database - CINAHL Complete
S50	TI ((food n2 (purchas* or select*))) OR AB ((food n2 (purchas* or select*)))	Search modes - Boolean/Phrase	Interface - EBSCOhost Research Databases Search Screen - Advanced Search Database - CINAHL Complete

S51	S22 OR S23 OR S24 OR S25 OR S26 OR S27 OR S28 OR S29 OR S30 OR S31 OR S32 OR S33 OR S34 OR S35 OR S36 OR S37 OR S38 OR S39 OR S40 OR S41 OR S42 OR S43 OR S44 OR S45 OR S46 OR S47 OR S48 OR S49 OR S50	Search modes - Boolean/Phrase	Interface - EBSCOhost Research Databases<br>Search Screen - Advanced Search<br>Database - CINAHL Complete
S52	TI (((intervention or app or e-mail or email or m-health or e-health or digital health or online or web*) n5 (adopt* or uptake or retention or maintenance or participat* or engag* or usage attrition or usage or adher*))) OR AB (((intervention or app or e-mail or email or m-health or e-health or digital health or online or web*) n5 (adopt* or uptake or retention or maintenance or participat* or engag* or usage attrition or usage or adher*)))	Search modes - Boolean/Phrase	Interface - EBSCOhost Research Databases<br>Search Screen - Advanced Search<br>Database - CINAHL Complete
S53	TI ((process n1 (metric* or evaluation))) OR AB ((process n1 (metric* or evaluation)))	Search modes - Boolean/Phrase	Interface - EBSCOhost Research Databases<br>Search Screen - Advanced Search<br>Database - CINAHL Complete
S54	TI login* OR AB login*	Search modes - Boolean/Phrase	Interface - EBSCOhost Research Databases<br>Search Screen - Advanced Search<br>Database - CINAHL Complete
S55	TI (page* n3 view*) OR AB (page* n3 view*)	Search modes - Boolean/Phrase	Interface - EBSCOhost Research Databases<br>Search Screen - Advanced Search<br>Database - CINAHL Complete
S56	TI (((module* or session*) n1 complet*))	Search modes - Boolean/Phrase	Interface - EBSCOhost Research Databases<br>Search Screen - Advanced Search<br>Database - CINAHL Complete

	OR AB (((module* or session*) n1 complet*))		
S57	TI (visit* n3 website*) OR AB (visit* n3 website*)	Search modes - Boolean/Phrase	Interface - EBSCOhost Research Databases<br>Search Screen - Advanced Search<br>Database - CINAHL Complete
S58	TI (time n3 online) OR AB (time n3 online)	Search modes - Boolean/Phrase	Interface - EBSCOhost Research Databases<br>Search Screen - Advanced Search<br>Database - CINAHL Complete
S59	TI ((user n3 (engag* or experience* or enjoyment or attention or interest or affect or immersion or involvement))) OR AB ((user n3 (engag* or experience* or enjoyment or attention or interest or affect or immersion or involvement)))	Search modes - Boolean/Phrase	Interface - EBSCOhost Research Databases<br>Search Screen - Advanced Search<br>Database - CINAHL Complete
S60	TI usability OR AB usability	Search modes - Boolean/Phrase	Interface - EBSCOhost Research Databases<br>Search Screen - Advanced Search<br>Database - CINAHL Complete
S61	S52 OR S53 OR S54 OR S55 OR S56 OR S57 OR S58 OR S59 OR S60	Search modes - Boolean/Phrase	Interface - EBSCOhost Research Databases<br>Search Screen - Advanced Search<br>Database - CINAHL Complete
S62	(MH "Cellular Phone") OR (MH "Smartphone") OR (MH "Mobile Applications")	Search modes - Boolean/Phrase	Interface - EBSCOhost Research Databases<br>Search Screen - Advanced Search<br>Database - CINAHL Complete
S63	TI "mobile device*" OR AB "mobile device*"	Search modes - Boolean/Phrase	Interface - EBSCOhost Research Databases<br>Search Screen - Advanced Search<br>Database - CINAHL Complete
S64	(MH "Virtual Reality") OR "Virtual reality"	Search modes - Boolean/Phrase	Interface - EBSCOhost Research Databases<br>Search Screen - Advanced Search<br>Database - CINAHL Complete
S65	(MH "Online Systems") OR "online"	Search modes - Boolean/Phrase	Interface - EBSCOhost Research Databases<br>Search Screen - Advanced Search<br>Database - CINAHL Complete
S66	(MH "Internet") OR "Internet"	Search modes - Boolean/Phrase	Interface - EBSCOhost Research Databases<br>Search Screen - Advanced Search<br>Database - CINAHL Complete

S67	TI (web n1 based) OR AB (web n1 based)	Search modes - Boolean/Phrase	Interface - EBSCOhost Research Databases<br>Search Screen - Advanced Search<br>Database - CINAHL Complete
S68	TI ((world wide web or WWW or website*)) OR AB ((world wide web or WWW or website*))	Search modes - Boolean/Phrase	Interface - EBSCOhost Research Databases<br>Search Screen - Advanced Search<br>Database - CINAHL Complete
S69	(MH "Electronic Mail") OR "e-mail" or "email"	Search modes - Boolean/Phrase	Interface - EBSCOhost Research Databases<br>Search Screen - Advanced Search<br>Database - CINAHL Complete
S70	TI (((mobile or cellular or cell or smart) n1 (phone* or telephone*))) OR AB (((mobile or cellular or cell or smart) n1 (phone* or telephone*)))	Search modes - Boolean/Phrase	Interface - EBSCOhost Research Databases<br>Search Screen - Advanced Search<br>Database - CINAHL Complete
S71	TI ((e-health or ehealth or "electronic health")) OR AB ((e-health or ehealth or "electronic health"))	Search modes - Boolean/Phrase	Interface - EBSCOhost Research Databases<br>Search Screen - Advanced Search<br>Database - CINAHL Complete
S72	TI (("m-health" or mhealth or "mobile health")) OR AB (("m-health" or mhealth or "mobile health"))	Search modes - Boolean/Phrase	Interface - EBSCOhost Research Databases<br>Search Screen - Advanced Search<br>Database - CINAHL Complete
S73	TI ((("digital health" or digital) n3 intervention*)) OR AU ((("digital health" or digital) n3 intervention*))	Search modes - Boolean/Phrase	Interface - EBSCOhost Research Databases<br>Search Screen - Advanced Search<br>Database - CINAHL Complete
S74	TI ((interactive n1 ((health n1 communicat*) or video* or technolog* or multimedia))) OR AB ((interactive n1 ((health n1 communicat*) or video* or technolog* or multimedia)))	Search modes - Boolean/Phrase	Interface - EBSCOhost Research Databases<br>Search Screen - Advanced Search<br>Database - CINAHL Complete

S75	TI (((chat n1 room*) or chatroom*)) OR AB (((chat n1 room*) or chatroom*))	Search modes - Boolean/Phrase	Interface - EBSCOhost Research Databases Search Screen - Advanced Search Database - CINAHL Complete
S76	(MH "User-Computer Interface")	Search modes - Boolean/Phrase	Interface - EBSCOhost Research Databases Search Screen - Advanced Search Database - CINAHL Complete
S77	TI "human computer interaction*" OR AB "human computer interaction*"	Search modes - Boolean/Phrase	Interface - EBSCOhost Research Databases Search Screen - Advanced Search Database - CINAHL Complete
S78	TI (phone n1 app*) OR AB (phone n1 app*)	Search modes - Boolean/Phrase	Interface - EBSCOhost Research Databases Search Screen - Advanced Search Database - CINAHL Complete
S79	S62 OR S63 OR S64 OR S65 OR S66 OR S67 OR S68 OR S69 OR S70 OR S71 OR S72 OR S73 OR S74 OR S75 OR S76 OR S77 OR S78	Search modes - Boolean/Phrase	Interface - EBSCOhost Research Databases Search Screen - Advanced Search Database - CINAHL Complete
S80	S21 OR S51	Search modes - Boolean/Phrase	Interface - EBSCOhost Research Databases Search Screen - Advanced Search Database - CINAHL Complete
S81	S61 AND S79 AND S80	Limiters - English Language; Human Search modes - Boolean/Phrase	Interface - EBSCOhost Research Databases Search Screen - Advanced Search Database - CINAHL Complete

CENTRAL

Trials matching on 'Smartphone* or “cell phone*” or “smart phone*” or “mobile device*” or “mobile app*” or “virtual reality” or online or internet or “web based” or “world wide web” or www or website* or email* or “e-mail*” or “electronic mail*” or “e-health” or ehealth or “electronic health” or “m-health” or mhealth or “mobile health” or ((digital health or digital) near/3 intervention*) or interactive or video* or technolog* or multimedia or “chat room*” or chatroom* or “phone app*” or “user computer interface” or “human computer interaction*” in Title Abstract Keyword AND Usability or ((intervention or app or e-mail or email or m-health or e-health or digital health or online or web*) near/5 (adopt* or uptake or retention or maintenance or participat* or engag* or usage attrition or usage or adher*)) or “process metric*” or “process evaluation*” or login* or (page* near/3 view*) or ((module* or session*) near/1 complet*) or (visit* near/3 website*) or (time near/3 online) or (user near/3 (engag* or experience* or enjoyment or attention or interest or affect or immersion or involvement)) in Title Abstract Keyword AND 'exercis* or gymnastics or “physical conditioning” or running or swimming or walking or “motor activity” or “physical education and training” or “physical fitness” or “cardiorespiratory fitness” or “life style” or lifestyle or “healthy ag?ing” or “healthy diet” or sport* or “physical activit*” or “physical inactivit*” or sedentary or “fitness class*” or “fitness regime*” or “fitness program*” or diet or nutrition* or “child nutrition science*” or fruit* or vegetable* or canteen* or “food service*” or calorie* or “energy intake” or “energy density” or “feeding behavio*” or “dietary intake*” or food or “carbonated beverage*” or “soft drink*” or soda or “sweetened drink” or “dietary fat*” or confectionar* or “school lunch*” or “school meal*” or “feeding program*” or cafeteria* in Title Abstract Keyword - (Word variations have been searched)'

Academic Search Ultimate

exercis* or gymnastics or “physical conditioning” or running or swimming or walking or “motor activity” or “physical education and training” or “physical fitness” or “cardiorespiratory fitness” or “life style” or lifestyle or “healthy ag?ing” or “healthy diet” or sport* or “physical activit*” or “physical inactivit*” or sedentary or “fitness class*” or “fitness regime*” or “fitness program*” or diet or nutrition* or “child nutrition science*” or fruit* or vegetable* or canteen* or “food service*” or calorie* or “energy intake” or “energy density” or “feeding behavio*” or “dietary intake*” or food or “carbonated beverage*” or “soft drink*” or soda or “sweetened drink” or “dietary fat*” or confectionar* or “school lunch*” or “school meal*” or “feeding program*” or cafeteria*

Usability or ((intervention or app or e-mail or email or m-health or e-health or digital health or online or web*) n5 (adopt* or uptake or retention or maintenance or participat* or engag* or usage attrition or usage or adher*)) or “process metric*” or “process evaluation*” or login* or (page* n3 view*) or ((module* or session*) n1 complet*) or (visit* n3 website*) or (time n3 online) or (user n3 (engag* or experience* or enjoyment or attention or interest or affect or immersion or involvement))

Smartphone* or “cell phone*” or “smart phone*” or “mobile device*” or “mobile app*” or “virtual reality” or online or internet or “web based” or “world wide web” or www or website* or email* or “e-mail*” or “electronic mail*” or “e-health” or ehealth or “electronic health” or “m-health” or mhealth or “mobile health” or ((digital health or digital) n3 intervention*) or interactive or video* or technolog* or multimedia or “chat room*” or chatroom* or “phone app*” or “user computer interface” or “human computer interaction*”

Scopus

TITLE (exercis* OR gymnastics OR "physical conditioning" OR running OR swimming OR walking OR "motor activity" OR "physical education and training" OR "physical fitness" OR "cardiorespiratory fitness" OR "life style" OR lifestyle OR "healthy ag?ing" OR "healthy diet" OR sport* OR "physical activit*" OR "physical inactivit*" OR sedentary OR "fitness class*" OR "fitness regime*" OR "fitness program*" OR diet OR nutrition* OR "child nutrition science*" OR fruit* OR vegetable* OR canteen* OR "food service*" OR calorie* OR "energy intake" OR "energy density" OR "feeding behavio*" OR "dietary intake*" OR food OR "carbonated beverage*" OR "soft drink*" OR soda OR "sweetened drink" OR "dietary fat*" OR confectionar* OR "school lunch*" OR "school meal*" OR "feeding program*" OR cafeteria*) AND ABS (usability OR ((intervention OR app OR e-mail OR email OR m-health OR e-health OR digital AND health OR online OR web*) AND (adopt* OR uptake OR retention OR maintenance OR participat* OR engag* OR usage AND attrition OR usage OR adher*)) OR "process metric*" OR "process evaluation*" OR login* OR (page* AND view*) OR ((module* OR session*) AND complet*) OR (visit* AND website*) OR (time AND online) OR (user AND (engag* OR experience* OR enjoyment OR attention OR interest OR affect OR immersion OR involvement))) AND ABS (smartphone* OR "cell phone*" OR "smart phone*" OR "mobile device*" OR "mobile app*" OR "virtual reality" OR online OR internet OR "web based" OR "world wide web" OR www OR website* OR email* OR "e-mail*" OR "electronic mail*" OR "e-health" OR ehealth OR "electronic health" OR "m-health" OR mhealth OR "mobile health" OR (digital AND intervention*) OR ((interactive OR video* OR technolog* OR multimedia) AND health) OR "chat room*" OR chatroom* OR "phone app*" OR "user computer interface" OR "human computer interaction*") AND (LIMIT-TO (LANGUAGE , "English ")) AND (LIMIT-TO (EXACTKEYWORD , "Human ") OR LIMIT-TO (EXACTKEYWORD , " Humans "))
