# Supplementary material for: Associations between Digital Health Intervention Engagement and Dietary Intake: A Systematic Review
Source: Nutrients. 2021 Sep 20;13(9):3281. doi: 10.3390/nu13093281 (PMC8470016; doi:10.3390/nu13093281)
Supplement: Supplementary file 1 [file nutrients-13-03281-s001.zip › Supplementary File 3 - Newcastle Ottawa Quality Assessment Tool.pdf]

SUPPLEMENTARY FILE – Newcastle Ottawa Scale Cohort and Cross Sectional Studies

Newcastle-Ottawa Quality Assessment Form for Cohort Studies

Note: A study can be given a maximum of one star for each numbered item within the Selection and Outcome categories. A maximum of two stars can be given for Comparability.

Selection

1. Representativeness of the exposed cohort
   a) Truly representative ***(one star)***
   b) Somewhat representative ***(one star)***
   c) Selected group
   d) No description of the derivation of the cohort

2. Selection of the non-exposed cohort
   a) Drawn from the same community as the exposed cohort ***(one star)***
   b) Drawn from a different source
   c) No description of the derivation of the non exposed cohort

3. Ascertainment of exposure
   a) Secure record (e.g., website analytics) ***(one star)***
   b) Structured interview ***(one star)***
   c) Written self report
   d) No description
   e) Other

4. Demonstration that outcome of interest was not present at start of study
   a) Yes ***(one star)***
   b) No

Comparability

1. Comparability of cohorts on the basis of the design or analysis controlled for confounders
   a) The study controls for age, sex and marital status ***(one star)***
   b) Study controls for other factors (list) _________________________________ ***(one star)***
   c) Cohorts are not comparable on the basis of the design or analysis controlled for confounders

Outcome

1. Assessment of outcome
   a) Independent blind assessment ***(one star)***
   b) Record linkage ***(one star)***
   c) Self report
   d) No description
   e) Other

2. Was follow-up long enough for outcomes to occur
   a) Yes ***(one star)***
   b) No
   Indicate the median duration of follow-up and a brief rationale for the assessment above:____________________

3. Adequacy of follow-up of cohorts
   a) Complete follow up- all subject accounted for ***(one star)***
   b) Subjects lost to follow up unlikely to introduce bias- number lost less than or equal to 20% or description of those lost suggested no different from those followed. ***(one star)***

c) Follow up rate less than 80% and no description of those lost
d) No statement

SCORING
Thresholds for converting the Newcastle-Ottawa scales to AHRQ standards (good, fair, and poor):
Good quality: 3 or 4 stars in selection domain AND 1 or 2 stars in comparability domain AND 2 or 3 stars in outcome/exposure domain
Fair quality: 2 stars in selection domain AND 1 or 2 stars in comparability domain AND 2 or 3 stars in outcome/exposure domain
Poor quality: 0 or 1 star in selection domain OR 0 stars in comparability domain OR 0 or 1 stars in outcome/exposure domain

---

NEWCASTLE - OTTAWA QUALITY ASSESSMENT SCALE adapted for cross sectional studies

Selection: (Maximum 5 stars)

1. Representativeness of the sample:
   - a) Truly representative of the average in the target population. i.e. all subjects or random sampling ***(one star)***
   - b) Somewhat representative of the average in the target population. i.e.(non-random sampling ***(one star)***
   - c) Selected group of users.
   - d) No description of the sampling strategy.
2. Sample size:
   - a) Justified and satisfactory. ***(one star)***
   - b) Not justified.
3. Non-respondents:
   - a) Comparability between respondents and non-respondents characteristics is established, and the response rate is satisfactory. ***(one star)***
   - b) The response rate is unsatisfactory, or the comparability between respondents and non-respondents is unsatisfactory.
   - c) No description of the response rate or the characteristics of the responders and the non-responders.
4. Ascertainment of the exposure (risk factor):
   - a) Validated measurement tool. ***(two stars)***
   - b) Non-validated measurement tool, but the tool is available or described. ***(one star)***
   - c) No description of the measurement tool.

Comparability: (Maximum 2 stars)

1. The subjects in different outcome groups are comparable, based on the study design or analysis. Confounding factors are controlled.
   - a) The study controls for the most important factor (select one). ***(one star)***
   - b) The study control for any additional factor. ***(one star)***

Outcome: (Maximum 3 stars)

1. Assessment of the outcome:
   - a) Independent blind assessment. ***(two stars)***
   - b) Record linkage. ***(two stars)***
   - c) Self report. ***(one star)***
   - d) No description.
2. Statistical test:
   - a) The statistical test used to analyze the data is clearly described and appropriate, and the measurement of the association is presented, including confidence intervals and the probability level (p value). ***(one star)***
   - b) The statistical test is not appropriate, not described or incomplete.

This scale has been adapted from the Newcastle-Ottawa Quality Assessment Scale for cohort studies to perform a quality assessment of cross-sectional studies for the systematic review, “Are Healthcare Workers’ Intentions to Vaccinate Related to their Knowledge, Beliefs and Attitudes? A Systematic Review”. We have selected age and gender as the most important for comparability, given these have been shown to be important prognostic factors influencing engagement. In the adapted scale, one star was assigned for self-reported outcomes.
