# Supplementary figures and images for: Associations between Digital Health Intervention Engagement and Dietary Intake: A Systematic Review
Source: Nutrients. 2021 Sep 20;13(9):3281. doi: 10.3390/nu13093281 (PMC8470016; doi:10.3390/nu13093281)

Figure S1. PRISMA Flow diagram

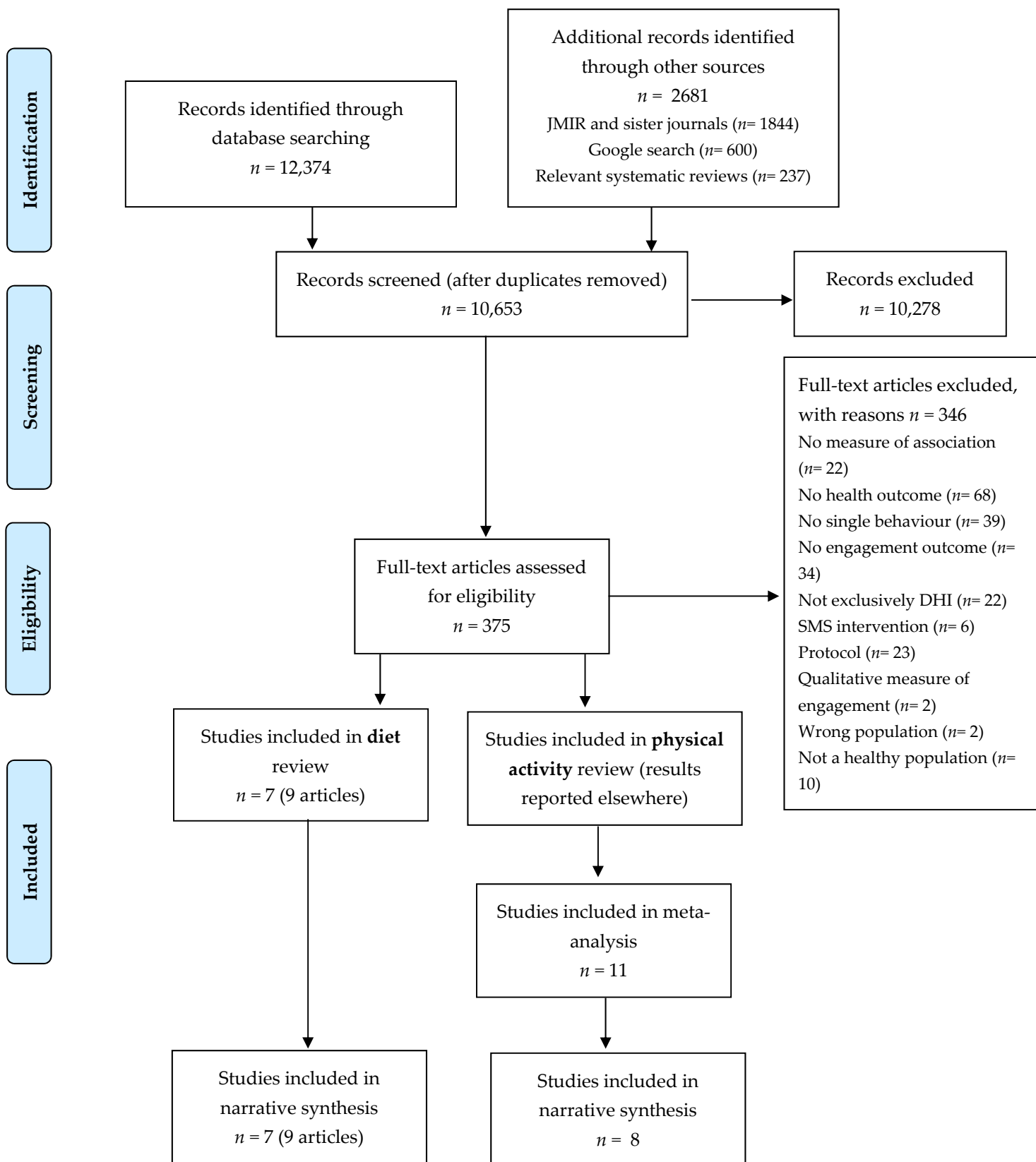

Supplement: Supplementary file 1 [file nutrients-13-03281-s001.zip › Supplementary File 2 - PRISMA flow diagram.pdf]
